# The Detection of Early Changes in Inflammatory Response After Pulmonary Vein Isolation in Patients with Paroxysmal Atrial Fibrillation Can Predict Late Atrial Fibrillation Recurrence

**DOI:** 10.3390/jcm14113874

**Published:** 2025-05-30

**Authors:** Ana Lanca Bastiancic, Ivana Grgic Romic, Snjezana Hrabric Vlah, Vlatka Sotošek, Marina Klasan, Petra Baumgartner, Mate Mavric, Sandro Brusich

**Affiliations:** 1 Clinic for Cardiovascular Diseases, Clinical Hospital Centre Rijeka, Tome Strizica 3, 51000 Rijeka, Croatia; ivanagr@medri.uniri.hr (I.G.R.); marina.klasan@kbc-rijeka.hr (M.K.); petra.baumgartner@medri.uniri.hr (P.B.); mate.mavric@medri.uniri.hr (M.M.); sandro.brusich@medri.uniri.hr (S.B.); 2 Department of Internal Medicine, Faculty of Medicine, University of Rijeka, B. Branchetta 20, 51000 Rijeka, Croatia; 3 Clinical Department of Laboratory Diagnostics, Clinical Hospital Centre Rijeka, Tome Strizica 3, 51000 Rijeka, Croatia; snjezana.hrabric.vlah@kbc-rijeka.hr; 4 Department of Anesthesiology, Reanimatology, Emergency and Intensive Care Medicine, Faculty of Medicine, University of Rijeka, 51000 Rijeka, Croatia; vlatkast@medri.uniri.hr; 5 Clinic for Anesthesia, Intensive Care Medicine and Pain Management, Clinical Hospital Center Rijeka, 51000 Rijeka, Croatia; 6 Department of Clinical Medical Studies II, Faculty of Health Studies, University of Rijeka, Viktora Cara Emina 2, 51000 Rijeka, Croatia

**Keywords:** atrial fibrillation, C-reactive protein, coronary sinus, pulmonary veins, transforming growth factor beta 1, tumour necrosis factor alpha

## Abstract

**Background**: Inflammation plays an important role in the initiation of atrial fibrillation (AF) and the development of fibrosis following pulmonary vein isolation (PVI). We aimed to investigate whether early post-PVI levels of C-reactive protein (CRP), white blood cells, tumour necrosis factor alpha (TNF-α) and transforming growth factor beta 1 (TGF-ß1) are associated with long-term arrhythmia recurrence. **Methods**: This prospective observational study included 48 patients with paroxysmal AF undergoing PVI. Peripheral venous blood samples were collected on the day of hospitalisation (T0), immediately after the procedure (T1) and after 24 h (T2), seven days (T3) and one month (T4) following the procedure. Blood samples were obtained from the coronary sinus (CS) before and after PVI. CRP levels, leukocyte (LKc) and neutrophile (Neu) counts were determined. TGF-β1 and TNF-α were analysed using the enzyme-linked immunosorbent assay (ELISA). After discharge, follow-up visits were scheduled at seven days and one-, three-, six-, nine- and twelve-months post-ablation, with 24 h Holter monitoring at each visit. **Results**: Patients were allocated into a recurrent and a non-recurrent group. Baseline characteristics did not differ between the groups, except for the duration of AF, which was found to be a significant arrhythmia recurrence predictor. Patients in the non-recurrent group had statistically significantly higher LKc at all time points, and Neu at T2 and T3. CRP and TGF-β1 concentrations were significantly higher in the non-recurrent group, while TNF-α concentration was significantly higher in the recurrent group at the T2 time point. Significantly higher concentrations of CS TNF-α at T1 and TGF-β1 at T0 and T1 were documented in the non-recurrent group. **Conclusions**: The study shows that an enhanced inflammatory response early after PVI, characterised by increased CRP, WBC and TGF-β1 levels, may play a protective role against late arrhythmia recurrence.

## 1. Introduction

Atrial fibrillation (AF) is the most common arrhythmia worldwide, associated with increased risk of stroke, heart failure and death [1,2]. It impairs quality of life and contributes to cognitive decline [2].

Haïssaguerre et al. have shown that ectopic beats originating from the pulmonary veins are a primary trigger for the spontaneous occurrence of cardiac arrhythmias, which can be effectively suppressed by electrical isolation of these areas [3].

Therefore, pulmonary vein isolation (PVI) has become the cornerstone of treatment for paroxysmal and persistent AF [2,3]. PVI is reasonably safe and is superior therapy to antiarrhythmic drugs for maintaining sinus rhythm and relieving symptoms [2]. Radiofrequency (RF) ablation and cryoablation are both well-established techniques that show comparable efficacy in the treatment of paroxysmal AF [4]. These procedures cause transient damage to the myocardium and trigger an inflammatory response that precedes the formation of fibrosis [5].

Inflammation is a recognised trigger for cardiac arrhythmias [6]. In this context, early arrhythmia episodes within the three-month post-PVI blanking period are not considered true recurrences, but part of the inflammatory process associated with the process of vein isolation [6,7]. However, some investigators have shown that for the same risk factors influencing the incidence of arrhythmias, early recurrence may only be an “early onset” of late recurrence, suggesting that early recurrence is an independent risk factor for late recurrence [8]. The rate of arrhythmia recurrence after PVI, including cryoablation and RF ablation, remains between 20 and 40% [9,10]. Since the introduction of PVI into clinical practice, numerous pre-ablation markers have been investigated as potential predictors of arrhythmia recurrence.

The levels of various inflammatory markers before ablation, including C-reactive protein (CRP), white blood cell count, and neutrophil-to-lymphocyte ratio, have been studied as potential predictors of arrhythmia recurrence after PVI, but the results are counterintuitive [11,12,13]. Their altered values have proven to be independent markers for the recurrence of cardiac arrhythmias after adjustment for various confounding variables [14,15]. Some studies suggest that elevated CRP levels before PVI may predict arrhythmia recurrence, suggesting that the suppression of inflammation may play a critical role in reducing arrhythmia episodes [15,16,17].

The role of transforming growth factor beta 1 (TGF-ß1) in predicting arrhythmia recurrence remains unclear and the results of studies are conflicting. Its pre-PVI levels may or may not be important in predicting arrhythmias in patients with paroxysmal AF [18,19,20,21,22,23]. Conversely, tumour necrosis factor alpha (TNF-α) levels have shown a correlation with left atrial diameter (LAD), but no clear association with arrhythmia recurrence [24].

The aim of this study was to investigate early post-PVI levels of inflammatory biomarkers, including CRP, neutrophils (Neu), leukocytes (Lkc), TNF-α and TGF-ß1, as potential predictors of arrhythmia recurrence.

## 2. Materials and Methods

### 2.1. Study Participants

**Figure 1:**
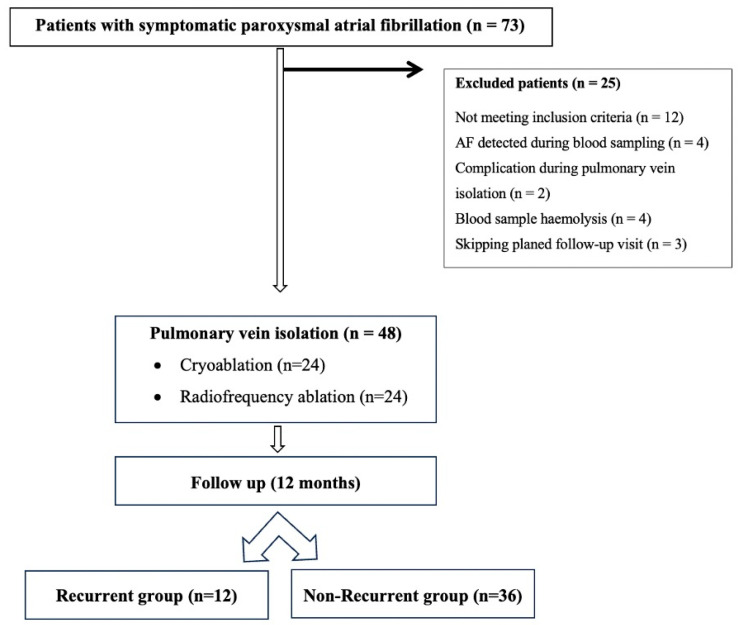
Flow diagram of the study.

This prospective observational study included 48 patients with paroxysmal symptomatic AF older than 18 years who were admitted to the Clinical Hospital Centre Rijeka, Rijeka, Croatia, for PVI between November 2022 and November 2023 (Figure 1). Patients were randomly selected for cryoballoon ablation or point-by-point RF ablation considering the results of the FIRE and ICE study [4].

Patients were excluded if they had a history of any AF ablation, were in AF at the time of enrolment, or had structural heart disease, chronic coronary syndrome, chronic inflammatory or autoimmune disease, chronic obstructive pulmonary disease or asthma, connective tissue disease, chronic kidney disease requiring haemodialysis, malignancies, pregnancy or acute inflammatory disease or had had recent surgery (within the past three months).

All included patients were anticoagulated according to the latest European Society of Cardiology (ESC) guidelines, with the last dose of a direct oral anticoagulant (DOAC) omitted on the morning of the procedure [2]. All selected patients gave written informed consent before inclusion in the study. Patients were excluded from the study if they experienced PVI complications, had haemolyzed blood samples, developed inflammatory disease at the time of scheduled follow-up visits, were in AF at the time of blood sampling, or failed to comply with the planned follow-up schedule. The study was approved by the Institutional Ethics Committee of the Clinical Hospital Centre Rijeka, Rijeka, Croatia, and the Ethics Committee of the Faculty of Medicine Rijeka, Rijeka, Croatia, and was conducted in accordance with the ethical principles of the Declaration of Helsinki.

### 2.2. Treatment Before Ablation

On admission, all patients underwent an initial examination that included a medical history, a physical examination and the recording of a 12-lead electrocardiogram (ECG).

#### 2.2.1. Blood Sampling

Peripheral venous blood samples were collected on the day of hospitalisation (T0) and at four additional specific time points: immediately after the procedure (T1), 24 h after the procedure (T2), on the seventh day (T3) and one month after the procedure (T4). Blood samples were also taken from the coronary sinus (CS) before and after PVI. All blood samples were taken while the patients were in sinus rhythm.

Blood samples were collected in standard plastic tubes: VACUETTE^®^ Tube with Sep/Clot Activator and K3EDTA (Greiner Bio-One GmbH, Kremsmünster, Austria) for serum and whole blood analysis, respectively. Each sample was labelled with a unique serial number and the corresponding sample type for proper identification. The samples were transported to the Clinical Department of Laboratory Diagnostics at the Clinical Hospital Center Rijeka, Rijeka, Croatia. Upon arrival, they were centrifuged for 10 min at 3500 rpm in a benchtop centrifuge to separate the serum. Routine laboratory tests were performed immediately, while the aliquots for biomarker analysis were stored at −20 °C until further processing.

#### 2.2.2. Biomarker Analysis

The serum biomarkers TGF-β1 and TNF-α were analysed using the enzyme-linked immunosorbent assay (ELISA) method. TGF-β1 concentrations were measured using the Human TGF-β1 ELISA Kit (BioVendor R&D Systems, Brno, Czech Republic). A standard curve was established by using the mean absorbance values of specific standard dilutions, each performed in duplicate, together with the corresponding concentrations. Samples were processed according to the manufacturer’s protocol and the TGF-β1 concentration was calculated from the standard curve. The measured concentrations were multiplied by the dilution factors to obtain the final values.

TNF-α concentration was measured using the Human TNF-α Ultrasensitive ELISA Kit (Invitrogen-ThermoFisher Scientific, Vienna, Austria).

Optical density measurements were performed using a HiPo MPP-96 microplate photometer (BioSan SIA, Riga, Latvia).

#### 2.2.3. Routine Laboratory Tests

The concentrations of N-terminal pro-B-type natriuretic peptide (NTproBNP) and troponin T (TnT) were measured using the electrochemiluminescence immunoassay (CLIA) method on a Cobas E601 analyser (Roche Diagnostics GmbH, Mannheim, Germany). TnT was determined using a high-sensitivity assay. The CRP level was determined on the Cobas C501 analyser using the turbidimetric method. The WBC count was determined using the DxH 800 analyser (Beckman Coulter, Brea, CA, USA).

#### 2.2.4. Echocardiographic Examination

Before the procedure, all patients underwent transthoracic echocardiography (TTE) using a GE system (GE HealthCare, Vivid E95 system, equipped with an M5Sc 1.4–4.6 MHz transducer, Boston, MA, USA), which included M-mode, 2D and Doppler imaging of sinus rhythm. The examination was performed by experienced physicians and all images were interpreted in accordance with the European Association of Cardiovascular Imaging (EACVI).

### 2.3. Procedure Details

The electrophysiologic study (EPS) and ablation were performed under light sedation using sufentanil and midazolam. The right femoral vein was accessed under ultrasound guidance (GE HealthCare, Vivid Iq Ultra Edition, Boston, MA, USA), and two sheaths (11F and 8F) were inserted for the intracardiac echocardiography (ICE) probe (AcuNav, Biosense Webster, Irvine, CA, USA) and for the decapolar CS catheter (WEBSTER Decapolar Catheter, Biosense Webster, Irvine, CA, USA). Transseptal puncture was ICE-guided and performed using a non-steerable transseptal sheath (HeartSpan, Merit Medical; HeartSpan Transseptal Needle, Merit Medical, Galway, Ireland). Periprocedurally, patients were anticoagulated with heparin, 5000 IU before and after puncture and thereafter in accordance with the activated clotting time (Medtronic, ACT Plus, Minneapolis, MN, USA), with the targeted ACT > 350 s.

#### 2.3.1. Radiofrequency Ablation

Radiofrequency (RF) ablation was performed using the CARTO 3 mapping system (Biosense Webster, Irvine, CA, USA). After transseptal puncture, the non-steerable transseptal sheath was exchanged for a steerable one (Agilis NxT, Abbot, St Jude Medical, Saint Paul, MN, USA; BF; S-, M- or L-curve), through which a multipolar “high density” mapping catheter (Pentaray NavECO, Biosense Webster, Irvine, CA, USA) was inserted. A three-dimensional map of the left atrium (LA) and a voltage map were created. Subsequently, the multipolar catheter was exchanged for a 3.5 mm irrigated tip ablation catheter (ThermoCool SmartTouch, Biosense Webster, Irvine, CA, USA) and a wide antral circumferential ablation (WACA) was performed using an ablation power of 50 W and an ablation index (AI) corresponding to the ablation region according to the CLOSE protocol (RF lesions by targeting an interlesion distance ≤ 6 mm and AI ≥ 400 at the posterior wall and ≥550 at the anterior wall) [25]. In case of dislocation, a new RF application reaching the AI target was applied. Exit block was confirmed by stimulating the veins within the ablation lines, and entry block was confirmed by positioning the multipolar catheter in each vein.

#### 2.3.2. Cryoablation

Cryoablation was performed using the CryoConsole cardiac ablation system (Medtronic, Minneapolis, MN, USA). After transseptal puncture, the non-steerable transseptal sheath was exchanged for a steerable one (Flex Cath Advance, Medtronic, Minneapolis, MN, USA, 15F) through which the cryoablation catheter (Artic Front Advance Pro, Medtronic, Minneapolis, MN, USA) and mapping circumferential catheter (Achieve Advance, Medtronic, Minneapolis, MN, USA) were inserted. Balloon position in the PVs was assessed by contrast administration before each freezing cycle. The duration of cryoapplication was the time to isolation (TTI; 180 to 240 s per operator discretion) and the temperature nadir guided. If no TTI and no temperature of −40 °C was reached for one minute, another lesion was applied [26]. The endpoint was the achievement of a bidirectional conduction block between LA and PVs and the disappearance of PV potential, which was confirmed with a circumferential mapping catheter.

### 2.4. Post-Procedure Management and Follow-Up

Anticoagulation therapy with DOACs was resumed the evening after the procedure and maintained for at least two months, in accordance with the patient’s CHA_2_DS_2_VASc score [2,27] and ESC guidelines [2]. Antiarrhythmic drugs (propafenone, amiodarone, flecainide) were prescribed as needed in highly symptomatic patients to maintain sinus rhythm during the blanking period, according to the ESC guidelines and You et al.’s study, which showed no correlation between the use of antiarrhythmic drugs after ablation (during the blanking period) and the prevention of late arrhythmia recurrences [28]. Then, 24 h after the procedure, a surface ECG and TTE were performed in all patients. Further laboratory tests were performed in parallel. After discharge, follow-up examinations were scheduled, the first after seven days and the second after one month, each with laboratory tests, 24 h Holter monitoring and echocardiography. Further follow-ups were scheduled at three, six, nine, and twelve months post-ablation, with 24 h Holter monitoring at each visit. Patients were instructed to visit the clinic for ECG recording if they experienced palpitations. According to the 2020 ESC guidelines [2], the recurrence of an arrhythmia was defined as an electrocardiographically documented episode lasting more than 30 s that occurred three months after PVI. Early nonsignificant recurrences were defined as episodes occurring in the first three months of the “blanking” period after ablation [2,29]. Based on the follow-up results, patients were divided into two groups: patients with arrhythmia recurrences and patients without arrhythmia recurrences.

### 2.5. Statistical Analysis

The statistical analysis was performed using Statistica 14.0.0. software (TIBCO Software Inc., San Ramon, CA, USA). The normality of the distribution was checked using the Kolmogorov–Smirnov test. The data had a non-normal distribution, and non-parametric statistical analysis was performed using the non-parametric Mann–Whitney U test to analyse the differences between the groups. The sample size was calculated based on our preliminary results for the groups. We set the type I error rate (alpha) to 0.05, the discriminatory power to 0.80, the RMSSE to 0.6, and the noncentrality parameter (delta) to 7.2. Of the above parameters, 23 patients were required in each group. Correlation was performed using Spearman’s rank correlation coefficient. A difference was considered statistically significant at a *p*-value < 0.05. All data are expressed as 25–75th percentile values.

## 3. Results

### 3.1. Demographic and Echocardiographic Characteristics

A total of 48 patients with paroxysmal symptomatic AF were included in the study (56% male, mean age 62.5 years). The patients were divided into two groups: the group with recurrent AF and the group without recurrence (Figure 1). The baseline characteristics of the patients did not differ between the groups, except for the duration of AF, which was found to be important for predicting late recurrences (Table 1).

There was no significant difference in echocardiographic parameters between the two groups (Table 2).

### 3.2. Changes in Laboratory Parameters and Cytokine Concentrations

#### 3.2.1. Peripheral Blood Samples

**Figure 2:**
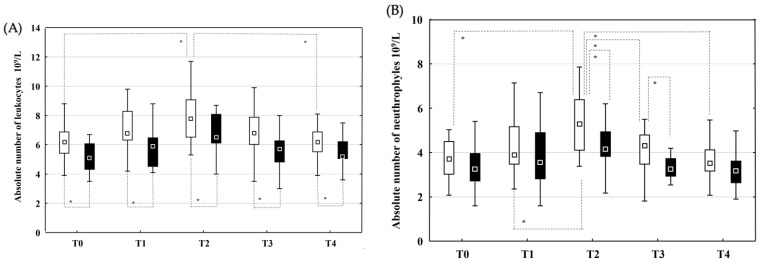

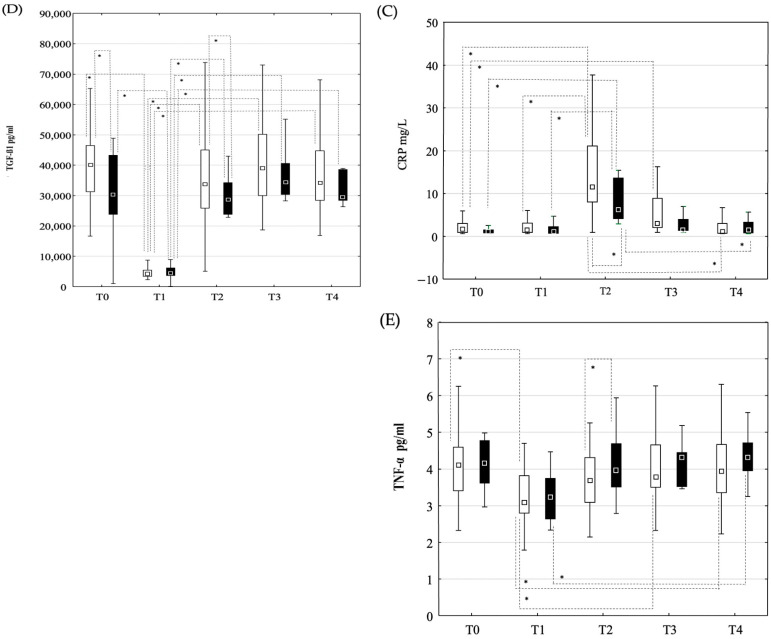
Dynamic changes in the absolute number of leukocytes (Lkc) and neutrophils (Neu), C-reactive protein (CRP), tumour necrosis factor alpha (TNF-α) and transforming growth factor beta 1 (TGF-ß1) concentrations. Absolute number of Lkc (**A**), Neu (**B**) and concentration of CRP (**C**), TGF-ß1 (**D**) and TNF-α (**E**) in peripheral venous blood in patients allocated to recurrence (■) and non-recurrence (□) groups at time points T0 (before PVI), T1 (immediately after PVI), T2 (24 h after PVI), T3 (7 days after PVI) and T4 (one month after PVI). Data are shown as medians and 25–75th percentiles. Level of statistical significance: * *p* < 0.05.

Patients in the non-recurrent group had statistically significantly higher LKc counts (Figure 2A) at all time points and higher Neu counts (Figure 2B) at the T2 and T3 time points. CRP concentrations were statistically higher in the non-recurrent group at T2 when compared to the recurrent group (Figure 2C). The absolute number of LKc (Figure 2A) and Neu (Figure 2B) increased significantly from T0 to T2 and then decreased until T4 in the non-recurrent group when compared to the recurrent group. CRP concentrations (Figure 2C) increased significantly from T0 to T2 and decreased by the T4 time point in both groups, with significantly higher values at T2 in the non-recurrent group (Figure 2C).

The concentration of TNF-α was significantly higher in the recurrent group than in the non-recurrent group at T2 (Figure 2E). There was a significant decrease in TNF-α concentration (Figure 2E) at time point T1 when compared to time point T0 and an increase in concentrations at time point T3 when compared to T1, and T4 when compared to the T3 time point in the non-recurrent group. The concentration of TGF-β1 (Figure 2D) was significantly higher in the non-recurrent group at time points T0 and T2 when compared to the same time points in the recurrent group. There was a significant decrease in TGF-β1 concentration (Figure 2D) in both groups at time point T1 compared to time point T0 and a significant increase at T2 time point when compared to T1.

**Figure 3:**
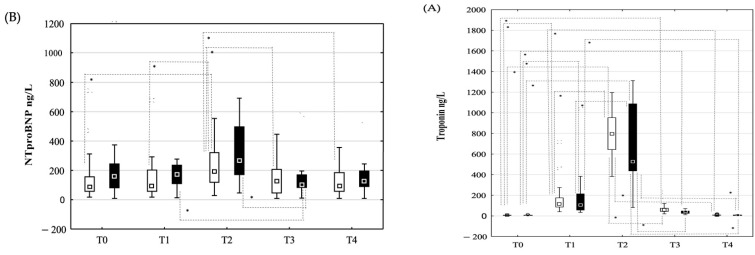
Dynamic changes in troponin T and NTproBNP concentrations. Concentration of troponin T (**A**) and NTproBNP (**B**) in peripheral venous blood in patients allocated to recurrence (■) and non- recurrence (□) groups at time points T0 (before PVI), T1 (immediately after PVI), T2 (24 h after PVI), T3 (7 days after PVI) and T4 (one month after PVI). Data are shown as medians (▫) and 25–75th percentiles. Level of statistical significance: * *p* < 0.05.

The dynamic changes in TnT (Figure 3A) and NTproBNP concentration (Figure 3B) did not differ significantly in both groups. In both groups, a significant increase in TnT concentration (Figure 3A) was observed at time points T1 and T2 compared to time point T0, followed by a decrease at time point T3 when compared to time point T2. NTproBNP concentration was also increased at time point T2 compared to time point T0 (Figure 3B) and then decreased at time point T4 when compared to time point T2 (Figure 3B).

#### 3.2.2. Blood Samples from the Coronary Sinus

**Figure 4:**
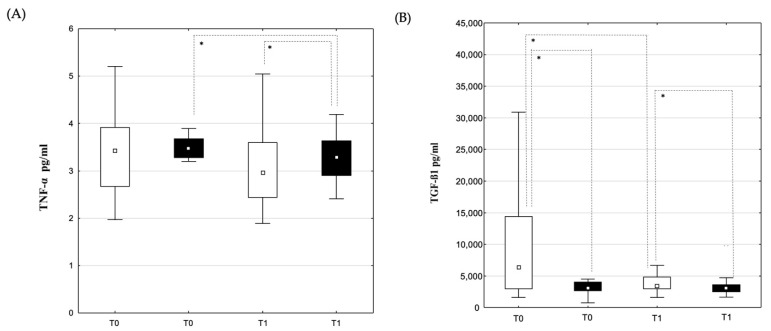
Dynamic changes in TNF-α and TGF-ß1 concentrations in coronary sinus (CS) samples. Concentration of TNF-α (**A**) and TGF-ß1 (**B**) in coronary sinus blood samples in patients allocated to recurrence (■) and non-recurrence (□) groups at time points T0 (before PVI) and T1 (immediately after PVI). Data are shown as medians and 25–75th percentiles. Level of statistical significance: * *p* < 0.05.

There was no significant difference between TNF-α concentrations measured before PVI (Figure 4A) in both groups. The statistically significant increase in TNF-α concentrations between time points T0 and T1 was documented in the recurrent group (Figure 4A), but not in the non-recurrent group. Comparing the recurrent and non-recurrent groups, TNF-α concentrations were significantly higher in the non-recurrent group at time point T1 (Figure 4A). TGF-ß1 concentrations before and after (Figure 4B) PVI were significantly higher in the non-recurrent group when compared to the recurrent group, and the decrease from time point T0 to T1 was pronounced in the non-recurrent group (Figure 4B), while there was no difference in the recurrent group.

## 4. Discussion

In the present study, our results suggest that lower CRP levels, together with lower Neu and LKc counts 24 h after ablation, are associated with a significantly higher degree of late arrhythmia recurrence, which was also particularly common in patients with lower TGF-β1 levels before and after PVI. An opposite high CS TGF-β1 level before, following a significant decrease after PVI, suggests that TGF-β1 upregulation after PVI can predict PVI success.

Guo et al. emphasised the central role of inflammation in both the development and progression of AF [30]. Several studies have shown that higher baseline CRP levels after electrical cardioversion are associated with an increased risk of arrhythmia recurrence [31,32,33]. However, the relationship between CRP concentration and the recurrence of AF after ablation is inconsistent and far from fully being understood. Contrary to our findings, Kurotobi [34] and Lellouche [35] et al. found that elevated baseline CRP concentrations were associated with an increased risk of early but not late recurrence of AF after ablation. However, Koyama et al. reported that there was no significant difference in baseline CRP concentrations between patients with and without AF recurrence within one month after ablation, although no long-term follow-up was performed [36]. Jun et al. showed a possible role of higher baseline CRP concentrations in predicting AF recurrence during long-term follow-up [37], while Verma et al. found that although LA scarring was correlated with higher CRP concentrations, baseline CRP concentrations did not differ significantly between patients with and without recurrence [38]. The different findings between these studies may be attributed to study design, patient characteristics, including age, duration of AF, LA size and the presence of risk factors and comorbidities [39].

Some studies suggest that inflammation plays a central role in the recurrence of arrhythmias by altering the distribution of fibroblasts and cardiomyocytes and promoting atrial remodelling and myopathy [40,41]. However, Fan et al. found no significant association between CRP concentrations (before and three months after PVI) and the recurrence of cardiac arrhythmias. They noted a rapid increase in CRP concentrations 24 h after PVI, reflecting an acute inflammatory response triggered by catheter ablation [42].

CRP is an important biomarker in fibrosis formation via several pathways [41,43,44]. It stimulates macrophages to produce interleukin (IL)-1 and TNF, both of which regulate fibroblast activation, angiogenesis and extracellular matrix deposition and promote scar tissue formation [41]. CRP also upregulates the profibrogenic cytokine IL-6, which has been investigated as an attractive therapeutic target for fibrosis prevention [43]. Zi Li et al. showed that CRP promotes inflammation and fibrosis formation by enhancing the activation of NF-κB and TGF-β/Smad signalling pathways [44]. Based on these mechanisms, low CRP concentrations may lead to a reduced inflammatory response and consequently to inadequate fibrosis formation after ablation.

TNF-α, a proinflammatory mediator known to promote fibrosis through direct effects on structural and immune cells [45], was significantly lower 24 h after PVI in the non-recurrent group. Previous studies linked high extracellular TNF-α levels to non-surviving patients with septic shock, cachexia and death in HIV patients, whereas intracellular TNF-α induces an NF-κB gene activation cascade that promotes fibrosis formation [45,46,47]. Accordingly, we hypothesise that the lower levels in the non-recurrent group are related to pronounced profibrotic activation and scarring. Recent studies have shown that fibroblast cultures with TNF-α or IL-13 induce fibrogenesis characterised by the progressively increased formation of type III and type IV collagen, and that TGF-β-1 co-stimulation enhances these effects [48].

TGF-β1 plays a central role in the fibroproliferative signalling pathway of atrial fibrosis and is therefore considered the most promising profibrotic biomarker for predicting the recurrence of AF after PVI [19,20]. Our study emphasises the importance of TGF β1 concentrations in predicting late arrhythmia recurrence. Patients in the non-recurrent group had significantly higher TGF β1 concentrations before and after PVI. Many studies have investigated the role of TGF-β1 in the initiation of AF and recurrence after PVI, with quite contradictory results [18,19,20,21,22,23]. TGF-β1 acts through multiple pathways, including SMAD, endothelial-to-mesenchymal transition (EndMT) and CD 44, and leads to the deposition of collagen I and III, resulting in atrial remodelling [20]. It is upregulated by angiotensin II in myofibroblasts and cardiac fibroblasts, and the use of angiotensin II receptor blockers has been shown to suppress the induction of TGF-β1 and prevent fibrosis [49,50]. Our study showed significantly increased TGF-β1 concentrations in the non-recurrent group before PVI, but previous studies showed no difference between these two groups in patients with paroxysmal AF, whereas a difference was found in patients with persistent AF [22]. TGF-β1 concentrations prior to PVI are influenced by AF and associated comorbidities causing oxidative stress and the activation of inflammatory processes with fibrosis formation, which was more pronounced in patients with persistent AF [21]. Plasma collagen, TGF-β1 mRNA and TGF-β1 concentrations gradually increase in patients with sinus rhythm, paroxysmal AF and persistent AF, suggesting that plasma TGF-β1 concentration correlates positively with the degree of atrial fibrosis [18]. Considering this and the fact that TGF-β1 may be the main cause of pulmonary vein (PV) arrhythmogenicity [49], TGF-β1 concentrations should be considered differently in patients with newly diagnosed, paroxysmal AF and long standing, persistent AF. In our study, the lower TGF-β1 levels before PVI in the recurrent group could be explained by the fact that it was activated in the inflammatory profibrotic process. The activated form of TGF-β1 is rarely detected as it has a very short half-life [50], which was also noted immediately after PVI when both groups showed a significant decrease in TGF-β1 concentrations. PVI injury leads to the conversion of cardiac fibroblasts into myofibroblasts by TGF-β1 stimulation [20,50]. This process is co-stimulated by inflammatory cytokines and chemokines produced by endothelial cells, which contribute to the recruitment of lymphocytes and macrophages with fibrotic effects in the process of endothelial-to-mesenchymal transition (EndMT) [20]. Two major functions of TGF-β1, wound healing and scar formation, are activated [10,20]. Our results showed a significantly higher release of TGF-β1 in the non-recurrent group 24 h after PVI, while myocardial injury estimated by TnT production did not differ between groups. Accordingly, we hypothesise that there were more non-endothelial cells in the recurrent group in the ablation region where sufficient inflammatory response and TGF-β1 activation were not achieved, resulting in poor fibrosis formation and possibly PV reconnection, which we could not confirm because patients were unwilling to undergo a second ablation. Wu et al. showed that vein reconnection was detected in patients with persistent AF and arrhythmia recurrence who underwent repeat ablation [22].

According to our results and the contradictory results of many other studies, TGF-β1 concentrations may be observed differently depending on the duration of AF and the ablation procedure performed, as the activation trigger is different, although the nature of fibrosis formation is similar but with different dynamics. The differing results from study to study may be due to the heterogeneity of the study population, the strategy of AF management and the TGF-β1 ELISA analysis performed, which could be performed with or without acidification [51].

We also measured TGF-β1 concentrations in the CS, which were significantly lower than peripheral levels, as in the small study by Zhao et al., who included 16 patients with chronic AF with a mean duration of 36 months and explained peripheral levels as a result of fibrosis of the multiple system, whereas the CS reflects a portion of ventricular venous blood [50]. Our study showed that TGF-β1 concentrations in the CS before PVI were significantly higher in the non-recurrent group, indicating that there was greater activation of TGF-β1 and remodelling of the atrium in the recurrent group. In the non-recurrent group, there was also a significant decrease in TGF-β1 concentrations in the CS after PVI, which we linked to the explicit promotion of wound healing by stimulating early responses such as inflammation and angiogenesis as well as later stages such as connective tissue formation and wound contraction [51]. There was no significant difference between TGF-β1 concentrations in the CS before and immediately after PVI in the recurrent group, supporting our hypothesis that the injury-induced inflammatory response was not sufficient to activate the profibrotic TGF-β1 cascade.

Although previous studies have emphasised the importance of anti-inflammation in preventing AF recurrence, anti-inflammatory therapies have not consistently improved outcomes after PVI [17]. Our results suggest that inflammation should not be overly suppressed in patients undergoing PVI. We hypothesise that this process is important for procedure associated fibrosis formation, and thus for the prevention of vein reconnections, which are known to be a primary mechanism for AF recurrence in patients with paroxysmal AF.

Among the baseline patient characteristics, the duration of AF before ablation was found to be the most important predictor of recurrence. Patients with a longer interval between diagnosis and ablation had a higher recurrence rate. These findings are consistent with the results of the Middelheim PVI registry, which concluded that earlier PVI is associated with better clinical outcomes [52].

There are several limitations of our study that need to be considered: We included a relatively small number of participants, but all consecutive patients who met the inclusion criteria—paroxysmal patients presenting in sinus rhythm for first AF ablation—were included. It is also important to emphasise that this was a single-centre prospective study involving only patients with structurally healthy hearts and paroxysmal AF, so our results cannot be extrapolated to patients with significant comorbidities and persistent AF. The detection of arrhythmias during follow-up was based on multiple 24 h Holter electro-cardiograms and patient-recognised symptomatic episodes of arrhythmia, whereas asymptomatic episodes may be misdiagnosed. Lastly, patients undergoing two different ablation techniques were included and in further multicentric study patients undergoing medical treatment could be included to determine this altered outcome.

## 5. Conclusions

Our study shows that the suppressed early inflammatory response after PVI, characterised by lower CRP levels, Neu and LKc counts, is associated with a higher degree of late AF recurrence. This is also supported by the finding of downregulation of TGF-β1 and TNF-α early after PVI, suggesting less pronounced profibrotic activation and scarring. The high TGF-β1 level in the CS before PVI and the subsequent significant decrease after PVI in the non-relapsing group suggests that TGF-β1 upregulation after PVI indicates profibrotic activation indexed by the procedure. In contrast, the fact that there was no difference between TGF-β1 levels in the CS before and immediately after PVI in the recurrent group supports our hypothesis that the injury-induced inflammatory response was not sufficient to activate the profibrotic TGF-β1 cascade. These results emphasise the complex role of inflammation and fibrosis formation after PVI and their potential role in predicting late AF recurrence.

## Figures and Tables

**Table 1:** Patients’ baseline characteristics in two groups.

Parameter	Non-Recurrence Group (*n* = 36)	Recurrence Group (*n* = 12)	*p* Value
Age (yr)	59.9 (53.5–68)	60 (58–66.5)	0.971
Gender (M/F)	22/14	5/7	0.40
Arterial hypertension	23/13	7/5	0.73
Diabetes mellitus type 2	2/34	2/10	0.22
Hyperlipoproteinemia	20/16	4/8	0.18
AF duration (mth)	40.5 (5–53.5)	58 (14.5–96)	0.05
Ablation (cryoablation/RF ablation)	19/17	5/7	0.504
Antiarrhythmic therapy during blanking period	23/13	8/4	0.86
BMI (kg/m^2^)	27.8 (24.9–31.2)	26.3 (24–30.13)	0.127
CHA_2_DS_2_VASc score	2 (0–5)	2 (0–4)	0.27
HAS BLED score	1 (0–1)	1 (0–2)	0.80
H_2_FpEFF score (%)	66.4 (60.25–78.6)	63.3 (52.35–78.7)	0.45

Data are shown as medians (25–75th percentile). AF—Atrial fibrillation, RF—radiofrequency, BMI—body mass index.

**Table 2:** Echocardiographic baseline findings in two groups.

Parameter	Non-Recurrence Group (n = 36)	Recurrence Group (n = 12)	*p* Value
LVEF (%)	60 (56–62)	58.5 (55.5–60)	0.066
LAD (mm)	42 (38–44)	39.5 (38–43.5)	0.060
LAVI (mL/m^2^)	25 (21–33.5)	30 (24–35)	0.35

Data are shown as medians (25–75th percentile). VEF—Left ventricle ejection fraction, LAD—left atrial diameter, LAVI—left atrial volume index.

## Data Availability

The data are available upon reasonable request.
